# The Health Advantage of a Vegan Diet: Exploring the Gut Microbiota Connection

**DOI:** 10.3390/nu6114822

**Published:** 2014-10-31

**Authors:** Marian Glick-Bauer, Ming-Chin Yeh

**Affiliations:** Nutrition Program, CUNY School of Public Health, Hunter College, City University of New York, 2180 Third Avenue, Room 614, New York, NY 10035, USA; E-Mail: mglickbauer@gmail.com

**Keywords:** vegan, vegetarian, gut microbiota, microflora, weight, obesity

## Abstract

This review examines whether there is evidence that a strict vegan diet confers health advantages beyond that of a vegetarian diet or overall healthy eating. Few studies include vegan subjects as a distinct experimental group, yet when vegan diets are directly compared to vegetarian and omnivorous diets, a pattern of protective health benefits emerges. The relatively recent inclusion of vegan diets in studies of gut microbiota and health allows us the opportunity to assess whether the vegan gut microbiota is distinct, and whether the health advantages characteristic of a vegan diet may be partially explained by the associated microbiota profile. The relationship between diet and the intestinal microbial profile appears to follow a continuum, with vegans displaying a gut microbiota most distinct from that of omnivores, but not always significantly different from that of vegetarians. The vegan gut profile appears to be unique in several characteristics, including a reduced abundance of pathobionts and a greater abundance of protective species. Reduced levels of inflammation may be the key feature linking the vegan gut microbiota with protective health effects. However, it is still unclear whether a therapeutic vegan diet can be prescribed to alter the gut microflora for long-term health benefits.

## 1. Introduction

As vegetarian diets have increased in popularity, veganism has gained recognition as a healthy and potentially therapeutic dietary choice [1]. A recent survey utilizing a food frequency questionnaire from 1475 participants, including 104 vegans, found that a vegan diet received the highest diet quality score as measured by the Healthy Eating Index 2010 and the Mediterranean Diet Score, while unrestricted omnivores received the lowest [2]. Vegan diets have gained acceptance as a dietary strategy for maintaining good health and managing disease conditions ranging from cardiovascular disease to cancer [1]. Vegan diets may prove useful as medical nutrition therapy in treating the conditions of metabolic syndrome, including obesity, diabetes and cardiovascular risk [3,4,5], and may confer protection against inflammatory conditions such as rheumatoid arthritis (RA) [6,7]. This review examines whether there is evidence that strict vegan diets confer protective benefits beyond that of vegetarian diets or overall healthy eating. Furthermore, the possibility that a vegan health advantage may be associated with a unique gut microbiota profile will be explored as a mechanism by which a vegan diet might be protective against metabolic and inflammatory disease states.

### 1.1. Are the Health Benefits of a Vegan Diet Unique?

Very few studies rigorously evaluate and compare omnivorous, vegetarian and vegan subjects as distinct experimental groups. It is thus difficult to discern whether the health advantages attributed to vegans could be generalized to all vegetarians or even to moderate meat eaters following a healthy diet. For example, Goff *et al.* [8] compared biochemical profiles of vegans and omnivores matched for gender, age and body mass index. The 21 vegans were found to have lower blood pressure, and lower fasting triacylglycerol and glucose concentrations than 25 omnivorous subjects, as well as a biochemical profile that was cardioprotective and beta-cell protective. Similarly, the health characteristics of 21 sedentary vegans who followed a long-term raw vegan diet were found to be comparable to that of endurance exercisers, with reduced BMI, lipids, lipoproteins, glucose, insulin, C-reactive protein, blood pressure and carotid artery intima-media thickness when compared to 21 sedentary subjects following a Western diet [9].

However, as these studies only compared vegans to omnivores, it is probable that a similar advantage may have been observed in vegetarian subjects as well. Vegetarian diets in general have been credited with improving insulin resistance [10], lowering diabetes risk [4,11], and lowering cardiometabolic disease risk [3]. A study of 170 vegetarian Buddhist monks and 126 omnivorous men found that the vegetarians had significantly lower BMI, blood pressure, and triglycerides as well as more favorable lipoprotein and apolipid profiles and lower predicted probability of coronary heart disease [12]. A study of 45 long-term (≥15 years) vegetarians found lower levels of oxidative stress, body fat and cholesterol than in 30 gender- and age-matched omnivores [13].

Even diets which allow moderate amounts of animal products may be protective against disease. A pooled analysis of five prospective cohort studies, involving 76,000 subjects found that both vegetarians and those who followed a “prudent” diet allowing small amounts of red meat benefited from a reduced risk of coronary heart disease and type 2 diabetes [14]. A retrospective cohort study utilizing a Taiwan longitudinal health checkup database of 93,209 participants (of which 1116 were vegan), found no decrease in metabolic syndrome with a vegan diet when compared to pescovegetarian, lactovegetarian and nonvegetarian diets [15]. These studies suggest that a vegan diet warrants further investigation to distinguish any uniquely protective effects not available from vegetarian or other less restrictive diets.

The Adventist Health Study-2 provides a unique opportunity to compare not only vegetarians to omnivores, but to distinguish between four types of vegetarian diet, designated as vegan, lacto-ovo vegetarian, pescovegetarian, and semi-vegetarian. While vegetarian diets in general were found to be beneficial when compared to omnivore diets, vegan diets appeared to confer a particular advantage in lowering odds ratios for developing type 2 diabetes [4,16], and all-cause mortality [17]. Hazard ratios for developing overall and female-specific cancers was similarly lower for those adhering to a vegan diet. Vegans in this population were found to have the lowest BMI when compared to all other diet types [18], although researchers noted some inadequate nutrient intake by the subjects following the strictest vegetarian diets. While Le and Sabaté [3] found that vegetarian diets in the Adventist cohort conferred protection against cardiovascular diseases, cardiometabolic risk factors, some cancers and total mortality, vegan diets in particular provided additional protection against obesity, hypertension, type 2 diabetes and cardiovascular mortality.

Other studies support the findings of the Adventist Health Study-2 population. A 74-week study on type 2 diabetes patients found that a low-fat vegan diet improved glycemia and plasma lipids significantly more than did a conventional diabetes diet [19,20]. A study of 23 overweight hyperlipidemic men and postmenopausal women who followed either a low-carbohydrate vegan diet or a high-carbohydrate lacto-ovo vegetarian diet for 7 months found that the vegan diet resulted in greater weight loss and decreases in LDL-cholesterol and triglycerides, and improved heart disease risk factors [5]. Thus in studies where vegans are compared to not only omnivores but to other vegetarians as well, a pattern of protective health benefits is emerging.

### 1.2. Link between Diet, Microbiota and Health

Recent gains in our understanding of the human gut microbiome has caused a shift in thinking, away from the concept of the “normal” gut, and toward an understanding of the complexity of our microbial communities and their functional properties in influencing health and disease [21]. The majority of microorganisms in the human intestine belong to the phyla Firmicutes (which includes *Clostridium*,* Enterococcus*, *Lactobacillus* and *Ruminococcus*) and Bacteroidetes (which includes *Bacteroides* and *Prevotella* in proportions determined in part by diet) [22,23] (See Table 1). Within these, three distinct enterotypes of the human gut microbiome were proposed by Arumugam [24]: (1) abundant *Bacteroides*; (2) few *Bacteroides* but abundant *Prevotella*; and (3) abundance of *Ruminococcus*. Each of these genera may be linked to distinct nutrient-metabolism functions and thus long-term dietary influences may play a key role. (See Power *et al*. [23] for a table of diet-microbe associations). Wu *et al.* [22] found two enterotypes associated with diet; the enterotype dominated by *Bacteroides* is adapted to diets high in protein and animal fats, while the *Prevotella* enterotype is associated with carbohydrate metabolism and a vegetarian diet. The National Institutes of Health Human Microbiome Project (HMP) found that bacteria from stool samples fell into four community types represented by a cluster of numerous taxa [25]. Others have concluded that although the HMP cohort is consistent with the two biome types found by Wu *et al*. [22], the data supports a gradient of microbial communities rather than distinct enterotypes [26,27].

Diet history is a factor that must be considered in the analysis of gut microbiota of any subjects [27]. Ruengsomwong *et al.* [28] found that among 13 Thai adults, non-vegetarians had a significantly higher abundance of *Bacteroides* while vegetarian subjects were enriched in the *Prevotella* genera. Microbiota dominated by Firmicutes (which includes *Ruminococcus*) have been strongly associated with a Western diet [29], and obesity [30], although there is debate over the significance of the relative ratios of Bacteroidetes and Firmicutes in lean and obese humans [31].

A study by De Filippo *et al*. [29] comparing European children to children in Burkina Faso, Africa found that the microbiome of European children consuming a Western diet is characterized by a predominance of the *Bacteroides* enterotype and a greater abundance of Gram-positive bacteria, mainly Firmicutes. The African microbiome, influenced by a high fiber, vegetarian diet is dominated by the *Prevotella* enterotype, with an overall enrichment in Gram-negative bacteria, mainly Bacteroidetes, and a corresponding depletion in Firmicutes. The African microbiome is also characterized by a higher microbial richness and diversity and a comparatively lower prevalence of pathogenic strains of Enterobacteriaceae. While diet plays a dominant role in shaping the gut microbiota in these disparate geographic communities, many other factors are thought to have an influence, including hygienic and sanitary conditions, prevalence of breast feeding, use of antibiotics and vaccines, ethnicity and geographic conditions [29].

Comparisons of dietary associations are complicated by the different methodologies used to study gut microbiota [32]. For example, both *Bacteroides* and *Prevotella* are characterized by polysaccharide-degrading bacteria [33]. As a group, *Bacteroides*-*Prevotella* have been shown to increase in abundance with a vegetarian or vegan diet using PCR-based DNA profiling techniques [34]. Thus the associations between diet and microbial communities may vary depending on whether researchers are conducting global assessments of gut microbiota or seeking greater taxonomic detail through DNA sequencing [32].

**Table 1:** Fecal microbiota community types based upon predominant taxa.

	Bacteroidetes		Firmicutes	
Arumugam *et al*. [24]	Enterotype1 ***Bacteroides***	Enterotype 2 ***Prevotella***	Enterotype 3 ***Ruminococcus***	
Wu *et al*. [22]	***Bacteroides***	***Prevotella***		
Huse *et al.* [26]	Gradient: ***Bacteroides/Ruminococcus*-*Prevotella***			
Jeffery *et al.* [27]	Gradient: ***Bacteroides*-*Prevotella***			
Ding & Schloss [25]	Community A * high Bacteroides * no Prevotella * no Ruminococcaceae	Community D * fewer *Bacteroides* than Community A or C * higher ***Prevotella***	Community C * lower Bacteroides * no Prevotella * higher ***Alistipes***, ***Faecalibacterium***, **Ruminococcaceae**	Community B * fewest Bacteroides * dominated by populations within **Firmicutes**

The HMP is exploring the diversity of our microbial communities to reveal both the richness and evenness of resident microbes [35]. While human illness was once approached with a narrow search for a single disease-causing microbe, it is now believed that the entire human gut microbiota exerts a direct influence on our health [21]. Gut dysbiosis, resulting from continued perturbations to the intestinal ecosystem, is implicated in disease states [36] as well as obesity [37]. Gut dysbiosis may contribute to a myriad of ailments in the host including allergies, celiac disease, gastric cancer, autism, obesity, anorexia, Irritable Bowel Disease, Crohn’s Disease, and type 2 diabetes (see Clemente *et al.* [36] for a table of implicated microbiota). It is likely that our host-gene-microbial interactions underlie complex autoimmune and inflammatory diseases, and that a better understanding of both the diversity and stability of our microbiota is necessary to identify personal disease phenotypes and improve medical treatments [38]. The inclusion of vegan diets in studies of gut microbiota and health allows us the opportunity to assess whether the vegan gut microbiota is distinct from that of vegetarians and omnivores, and whether the health advantages characteristic of a vegan diet may be partially explained by the associated microbiota profile.

## 2. Is the Vegan Gut Profile Unique?

As early as the 1970s researchers have been examining the role of diet on gut microflora by comparing diets high in meat (“mixed Western diet”) with vaguely defined non-meat diets. For example, Reddy *et al.* [39] found evidence that omnivores had an anaerobic microflora enriched in *Bacteroides*,* Bifidobacterium*,* Peptococcus* and *Lactobacillus* when compared to non-meat eaters. In the 1980’s vegan diets emerged as a defined subset of vegetarians worthy of study. While a few studies were able to take advantage of subjects who have been long-term vegans or lifelong vegetarians (such as the 7th Day Adventist research set), the majority of researchers relied upon experiments in which a short-term Western, vegetarian or vegan diet was imposed on subjects in order to measure the subsequent effect on gut microbiota or the products of microbial metabolism. This research has brought attention to the possibility of utilizing vegan or other therapeutic diets as medical intervention for a host of disease states.

van Faassen *et al*. [40] were among the first to include a vegan diet as one of their experimental diet conditions. The authors compared subjects on a 20-day vegan diet as compared to 20-day lacto-ovo vegetarian and mixed Western diets. While subjects were on the vegan diet they showed the lowest levels of fecal *Lactobacilli* and *Enterococci*, along with lower concentrations of bile acids, coprostanol, and coprostanol plus cholesterol. These results are notable in that the vegan diet produced a significantly different result than the lacto-ovo vegetarian diet, suggesting that the broad distinction between meat and non-meat diets seen in earlier studies was not sufficient and a focus on vegan diets in their own right is warranted.

In the last few years the research community has focused greater attention on vegan diets as a distinct experimental dietary condition. Recent studies have provided more detailed analysis of vegan, vegetarian and omnivore gut microbial profiles. Matijaŝić *et al*. [34] compared bacterial DNA from fecal samples of 20 vegans, 11 lacto-vegetarians and 29 omnivores who were comparable in gender, age, body mass and height, and found an association between diet type and bacterial community composition. PCR-based DNA profiling methods were used to quantify microbiota by group. Both the vegetarian and vegan subjects were associated with a higher ratio of the *Bacteroides-Prevotella* group when compared to omnivores. Both vegan and vegetarian subjects also had a lower ratio of *Clostridium* cluster XIVa when compared to omnivores, a result similarly noted by Kabeerdoss *et al*. [41], but in contrast to the findings of Kim *et al*. [33]. Only two distinctions were found between the vegans and the other vegetarians examined. Vegans had a higher ratio of *Faecalibacterium prausnitzii*, an anti-inflammatory bacterium and abundant butyrate producer in the class *Clostridia* (phylum Firmicutes), purported to play a protective role for colonocytes. Vegans also displayed a higher ratio of *C. clostridioforme* within the *C. coccoides* grouping.

A recent, large-scale study by Zimmer *et al*. [42] set out to distinguish the fecal microbiota profile of vegans (*n* = 105), from that of vegetarians (*n* = 144), and an equal number of controls consuming an omnivorous diet. Vegan and vegetarian subjects had adhered to their proclaimed diet for at least 4 weeks prior to the study. Vegan samples had significantly lower microbial counts than their omnivore counterparts for four bacterial taxa: *Bacteroides, Bifidobacterium*,* E. coli* and* Enterobacteriaceae*. Interestingly, the vegetarian sample also showed significantly reduced *Bacteroides* and *Bifidobacteria*, a result found 37 years earlier by Reddy [39]. It is important to note that Zimmer found that vegans and vegetarians were not significantly different from each other in these four taxa, nor did they differ in *Enterobacter*,* Enterococcus*,* Clostridium*,* Klebsiella* or* Lactobacillus*, when compared to each other or to the omnivore samples. Vegan diets are higher in carbohydrates and fiber than omnivores, and as such the vegan samples had significantly reduced stool pH than did controls. The lower pH was strongly correlated with reduced counts of *E. coli* and *Enterobacteriacea*, species which are not tolerant of the more acidic environment. The authors concluded that the gut microbiota and stool pH of vegetarians fell on a continuum between that of vegans and omnivores. These results suggest that the composition of the human gut is altered by diet along a continuum, with vegan diets being the most distinct from that of omnivores, but not necessarily significantly different from that of other vegetarians.

Interestingly, these findings are opposite those of other studies, although differing methodologies can make direct comparisons difficult. Matijaŝić *et al*. [34] found a higher ratio of the *Bacteroides-Prevotella* group associated with the vegetarian diets, while van Faassen *et al*. [40] found no effect of a vegan or vegetarian diet on *Bifidobacteria* and *Bacteroides*. Yet they did find lower counts of aerobic *Lactobacilli* and *Enterococci*, a result not found by Zimmer *et al*. [42]. Despite their often contradictory findings, these studies provide the first indication that there may be some distinction between the gut microbiota of a vegan and a vegetarian, although both diets appear to be responsible for significant shifts away from the omnivore’s gut profile. More large-scale studies are warranted to discern whether a distinction between vegans and vegetarians is legitimate, or whether life-long vegans can be compared with test subjects following a vegan diet for a short-term such as 20 days.

### Can a Dietary Shift Modify the Gut Profile?

It is not clear whether adopting a vegan diet as short-term medical nutrition therapy can lead to a gut profile or “enterotype” comparable to that of a long-term vegan. A new avenue of research is examining whether our intestinal microbiota is a stable ecosystem, resistant to change, or whether a novel diet can facilitate a rapid, adaptive shift in the intestinal microbial community [43,44,45,46]. There is evidence that the ratio of bacteria species in the intestines may shift in response to a novel diet, though this change in species may not constitute a larger shift from one enterotype to another. For example, Wu *et al.* [22] found that among 98 subjects, those who identified as vegetarian (including one vegan) showed enrichment in the *Prevotella* enterotype, while those who consumed meat as part of a Western diet were enriched in *Bacteroides*. Ten subjects who switched to a high-fat/low-fiber or low-fat/high-fiber diet displayed detectible changes in their microbiome composition within 24 hours, although their enterotype identity remained stable for the duration of the 10-day trial. Similarly, David *et al.* [43] reported a rapid adaptation of gut microbiota in response to a plant-based or animal-based diet, with adaptations to herbivorous and carnivorous functional profiles evident within five days on the new diet. Their data suggest that an animal-based diet has a greater impact on altering the gut microbiota than does a plant-based diet, as the animal-based diet increased levels of fecal bile acids, which increased the abundance of bile-tolerant organisms and decreased those species that metabolize dietary plant polysaccharides. Notably, the one life-long vegetarian among the 10 subjects showed a reduction in the genus *Prevotella* during the switch to an animal-based diet.

What is not clear from these short-term trials is whether dietary interventions, such as adopting a vegetarian or vegan diet, could stably switch subjects to a more beneficial enterotype and confer lasting health advantages. The human colonic microbiome is characterized by high resilience, a central concept in ecology whereby communities are able to withstand disturbance [21]. Faith *et al*. [44] followed 37 adult subjects and found that approximately 60% of their 200 microbial strains remained stable over the course of 5 years. Shared strains from family members suggest that hosts may retain their stable microbiota for decades. Rajilić-Stojanović *et al*. [46] followed 5 subjects for 8–12 years and concluded that although the relative abundance of intestinal bacteria species changed with diet, travel or antibiotic use, the core community of microbiota specific to each subject was conserved over many years. Similarly, Martínez *et al*. [45] followed three human subjects for one year and detected a stable core microbiota per individual, consisting of approximately 40 species (80% of the microbiota), which maintained persistent populations. The authors suggest that diet change may induce shifts within core members but the overall bacterial populations show resilience and return to baseline levels after the dietary intervention ceases. These findings call into question the efficacy of treating inflammatory diseases and metabolic disorders with short-term dietary interventions. More studies are warranted to determine if a long-term commitment to a plant-based therapeutic diet could more permanently alter an individual’s microbiota and confer health advantages.

## 3. Vegan Gut Microbiota May Be Protective against Metabolic Syndrome

Obesity is associated with an altered gut profile such that resident bacteria may be responsible for an increased capacity for energy harvest and a state of chronic, low-grade inflammation. This inflammation, in turn, can interfere with insulin signaling and results in the metabolic dysfunction found in obesity and type 2 diabetes [47,48]. Obesity has been linked to a decreased prevalence of Bacteroidetes (which includes both *Prevotella* and *Bacteroides*), and an increase in Firmicutes and Actinobacteria [49,50]. Ley *et al*. [30] found that a reduced calorie diet was sufficient to increase the relative abundance of Bacteroidetes relative to Firmicutes in obese subjects; the increase in Bacteroidetes and decrease in Firmicutes correlated with the percentage loss of body weight. In contrast, a balanced diet characterized by high consumption of fruits and vegetables and low consumption of meat leads to a highly diverse intestinal flora and a greater abundance of *Prevotella* over *Bacteroides* [49].

Interestingly, *F. prausnitzii*, the most abundant bacterium in the intestines of healthy adults and a member of the Firmicutes, is one of the species Matijaŝić *et al*. [34] noted was distinctly more prevalent in vegans than in vegetarians. *F. prausnitzii* appears to play a significant protective role in metabolic disease, with depressed levels associated with intestinal disorders, inflammation and obesity [51], and type 2 diabetes [52,53]. Remely *et al*. [52] found that the gut profile of diabetics differs from that of lean controls, and noted that *F. prausnitzii* was most abundant in lean controls but least abundant in type 2 diabetics, a finding that agrees with prior studies. The prevalence of this species in the gut has been linked to diet, specifically ingestion of the plant polysaccharide, inulin [51]. Intestinal microbiota are able to produce short chain fatty acids (SCFA), acetate, propionate, and butyrate, through metabolism of dietary fiber. A strong positive correlation has been found between *F. prausnitzii* and butyrate production in the gastrointestinal tract, suggesting that this species may be associated with higher fiber intake and reduced risk for cardiovascular disease, colon cancer, diabetes and obesity [54].

It is possible that the disproportionately high prevalence of this beneficial bacterium in the vegan gut is attributable to a high fiber diet. The role of dietary fiber needs to be examined in greater depth, beyond its mechanical effect of increasing stool bulk and speeding transit time. Dietary fiber also influences the intestinal environment by inhibiting pathogen adhesion, altering bacterial fermentation patterns and short chain fatty acid concentrations, modifying microbiota community profiles, and lowering stool pH [32,42]. De Filippo *et al*. [29] found a greater concentration of SCFA in fecal samples of children in rural Africa as compared to their European counterparts. The authors hypothesized that SCFA-producing bacteria are selected for by a diet high in plant polysaccharides and low in fat and sugar. Further research is warranted to determine if there is a link between a vegan diet, type and quantity of dietary fiber, an increased intestinal prevalence of *F. prausnitzii* and other SCFA-producing bacteria, and a corresponding reduction in the prevalence of obesity, type 2 diabetes, inflammation and intestinal disorders.

Inflammation may be the critical component linking gut microbiota with obesity as well as metabolic dysfunction and chronic disease [48,55]. Verdam *et al*. [56] found that among 28 subjects, the microbiota of the obese subjects, unlike that of the non-obese, was characterized by a reduced bacterial diversity, a decreased ratio of Bacteroidetes to Firmicutes, and an increased abundance of potentially inflammatory Proteobacteria. Moreover, the microbiota of obese subjects was associated with markers of local and systemic inflammation (fecal calprotectin and plasma C-reactive protein, respectively).

The role of a vegan diet in influencing obesity and inflammation was explored by Kim *et al*. [33], in their study of 6 obese subjects with diabetes and/or hypertension. Subjects who followed a vegan diet for one month, were found to have improved blood glucose levels and reduced body weight, as well as a reduction in triglycerides, total cholesterol, LDL-cholesterol and Hemoglobin A1c. The vegan diet therapy induced an altered gut microbiota by reducing the abundance of Firmicutes and increasing the abundance of Bacteroidetes significantly. Despite alterations in the Firmicutes-to-Bacteroidetes ratio, these changes did not result in a switch of the host’s enterotype, as both *Prevotella* and *Bacteroides* (responsible for degradation of plant polysaccharides) increased in response to a vegan diet. Notably, the vegan diet was associated with a decrease in pathobionts such as *Enterobacteriaceae*, a family of bacteria implicated in triggering low-grade inflammation, which Zimmer [42] also found were reduced in vegan subjects. The authors measured decreasing concentrations of the inflammation markers, fecal lipocalin-2 (Lcn-2), as the subjects progressed on their vegan diets, and concluded that the vegan diet directly reduced the population of pathobionts, thereby reducing inflammation and contributing to the improved glucose tolerance and lipid metabolism of their vegan subjects [33]. It has to be considered that the fiber content of vegan diets may play a significant role in regulating the inflammatory response. SCFA generated by gut microbiota can act as signaling molecules, activating G protein-coupled receptors and modulating the host’s inflammatory response [32]. Thus dietary fiber may be a key variable in any evaluation of vegan diets and inflammatory disease.

The most recent evidence that a vegan diet promotes a gut microbiota that directly reduces metabolic disease risk is the research linking diet to l-carnitine metabolism and atherosclerosis risk. Koeth *et al*. [57] found that microbial metabolism of dietary l-carnitine, a trimethylamine found in red meat, produces trimethylamine-*N*-oxide (TMAO), which has been shown to promote atherosclerosis. The authors had previously suggested a novel pathway by which gut microbiota metabolize choline/phosphatidyl choline to produce the intermediate compound trimethylamine (TMA). TMA is oxidized to TMAO which has been directly linked to atherosclerotic heart lesions [58], and has been show in rodent models to alter cholesterol and sterol metabolism, resulting in an increase in atherosclerosis [57]. Koeth *et al*. [57] sought to determine whether gut microbes are also responsible for TMAO production from dietary l-carnitine by testing vegans, vegetarians and omnivores with a “carnitine challenge”. 

The study found that TMAO production from dietary l-carnitine is dependent upon intestinal microbiota and the capacity to produce this TMAO was negligible in vegans. The preliminary carnitine challenge was conducted on one (*n* = 1) long-term (>5 years) vegan who displayed virtually no capacity to generate TMAO. When 23 additional vegans and vegetarians were examined and compared to 51 omnivorous subjects, fasting baseline TMAO levels were significantly lower in both the vegan and vegetarian subjects compared to omnivores. The long-term (>1 year) vegans and vegetarians who underwent the oral carnitine challenge displayed a reduced capacity to produce TMAO from dietary carnitine. Moreover, analysis of fecal samples from vegans/vegetarians (*n* = 23) and omnivores (*n* = 30) revealed that several bacterial genera were significantly associated with both plasma TMAO levels and dietary category (vegan/vegetarian *vs.* omnivore), suggesting that established dietary habits directly impact the ability to synthesize TMAO. Thus a direct link was established between diet type, intestinal bacterial taxa, plasma TMAO levels and associated risk for atherosclerosis.

It should be noted that in this study both vegans and vegetarians were found to be distinct from omnivores, yet not necessarily from each other. Vegans and vegetarians overall have been shown to have lower plasma carnitine concentrations than omnivores, and more efficient renal reabsorption of carnitine, particularly when dietary intake is limited [59]. Thus there appears to be a distinct vegetarian response to low dietary carnitine ingestion, characterized by both a greater efficiency at retaining carnitine, necessary for fuel metabolism in skeletal muscle, as well as a lack of the intestinal bacteria responsible for metabolizing dietary carnitine to the proatherosclerotic TMAO. This suggests that a vegan diet may be the best option to reduce proatherosclerotic TMAO and thus reduce CVD risk, but that similar benefits may be attained by a vegetarian diet. These findings support the idea of a continuum in which the vegan gut microbiota is most different from that of omnivores, yet not necessarily distinct from that of other vegetarians.

## 4. Vegan Gut Microbiota May Be Protective against Inflammatory Diseases

Studies have noted a link between vegan diets and protection against autoimmune diseases [60]. For example, an analysis of the Adventist cohort found that a vegan diet, but not a vegetarian diet, was associated with a lower risk of hypothyroid disease [60]. Ling and Hanninen [61] tested subjects on both a conventional Western diet and a raw vegan diet for one month and found that four fecal hydrolytic enzymes, associated with toxic and inflammatory products, diminished during the vegan diet. However, these changes in fecal urease, choloylglycine hydrolase, β-glucuronidase and β-glucosidase disappeared within two weeks of resuming a conventional diet. The authors attribute these reductions in fecal enzymes not only to the activity of bacteria during the dietary shift, but also to the high fiber content of the vegan diet which can affect fecal weight, transit time and bacterial metabolism.

More in-depth research has focused on vegan diets and the “extreme” raw vegan diet (the Living Food movement) as a promising treatment for rheumatoid arthritis (RA). This possibility that a vegan diet can induce a rapid change in gut profile was supported by studies of rheumatoid arthritis patients in which a one-month switch to a vegan diet was sufficient to significantly alter the fecal microflora, as determined by stool sample gas-liquid chromatography profiles of bacterial cellular fatty acids [7,62]. Peltonen *et al*. [63] conducted a study of 53 RA patients and found a significant change in intestinal flora after a one-year shift from a conventional diet to a vegan and then a lactovegetarian diet. They also noted a significant difference between the fecal flora of test subjects in the high improvement group and the low improvement group, suggesting a direct connection between gut profiles and levels of disease activity. 

To further test the role of fecal flora in diet-induced levels of rheumatoid arthritis activity, 43 RA patients were randomly assigned to either a raw vegan diet rich in *lactobacilli* or an omnivorous diet. After one month, there was a significant change in the fecal flora of the 18 subjects in the vegan diet group who completed the study; no such change was found in the omnivore control group. Importantly, the vegan diet also induced a decrease in disease activity in some of the RA patients, leading the authors to conclude that changes in the fecal flora are associated with diet-induced changes in disease activity [7].

Kjeldsen-Kragh [6] followed upon their work by putting rheumatoid arthritis patients on a fast followed by 3.5 months of a vegan diet, followed by a 9-month lactovegetarian diet. Subjects in the vegan/vegetarian diet group improved significantly over those maintained on an omnivorous diet. Similar to other studies, the authors found that subjects’ fecal flora during times of clinical improvement differed significantly from times of no or minor improvements. Others have found that a raw vegan diet rich in *lactobacilli* and fiber decreased symptoms of rheumatoid arthritis, suggesting that the probiotic *lactobacilli*, among other components of a raw vegan diet, may be helpful to RA patients [64,65,66]. However, while these studies effectively linked diet, microbiota profile and RA symptom severity, they either focused on vegans to the exclusion of vegetarians, or combined the diet types, thus allowing no way to discern if the improvement in RA symptoms in these studies can be attributed to a vegan diet *per se*, or whether comparably beneficial results could be attained by a less-restrictive and potentially easier-to-follow vegetarian diet.

Caution is warranted in interpreting the studies on vegan diets and RA. Although diet-induced modification in intestinal flora and a reduction in inflammation severity may be a contributing factor to the improvements seen in RA patients, it is important to note that other features of a vegan diet have been credited with alleviating RA symptoms among vegan diet adherents. These include an increase in fruit, vegetable and fiber intake, a reduction in saturated fat and caloric intake, altered antioxidant levels, weight loss, and a reduction in food allergies and intolerances [67,68].

## 5. Limitations

To the authors’ knowledge, this review is the first to assess the association between vegan diets, gut microbiota and human health outcomes. Nonetheless, several limitations should be noted. A substantial limitation of comparing studies of gut microbiota lies in the different methodologies utilized, as molecular biology has advanced rapidly in recent years, beyond conventional culturing techniques, to allow substantially greater detection of the number and diversity of human gut microbiota [32,50,69]. Moreover, comparisons of studies on vegan gut microbiota may be problematic in that fecal microbial profiles vary not only by diet, but by the subjects’ age, gender, and body mass [34]. Dietary fiber in particular may be a confounding variable when comparing vegan and omnivorous diets, given the interrelationship between fiber, microbial SCFA production, and the host’s inflammatory response [32].

A note of caution must be sounded in comparing health indices and disease risk of long-term vegans with those who follow a more typical Western diet, as lifestyle factors are significant confounding variables in these analyses. Among 100 vegans surveyed in the U.S., 47% cited health as the motive for their diet choice. This subset of vegans was associated with regular exercise, minimal alcohol and smoking practices, and frequent consumption of vegetables, nuts and grains [70,71]. The German Vegan study similarly found that, compared to the general population, vegans tend to refrain from smoking, consume limited alcohol, and engage in higher levels of physical activity [71]. Both studies found that vegans tend to have a low or normal BMI [70,71], which may be a factor in the lower rates of metabolic disease and cancer in this population. Thus more trials are warranted to determine the strength of the association between diet, microbiota profile, degree of inflammation and autoimmune response, and ultimately the manifestations of disease symptoms.

## 6. Conclusions 

The relationship between diet and the intestinal microbial profile appears to follow a continuum, with vegans displaying a gut microbiota most distinct from that of omnivores, but not always significantly different from that of vegetarians [34,42]. The vegan gut profile appears to be unique in several characteristics, including a reduced abundance of pathobionts, including *Enterobacteriacea* [33,42], and a greater abundance of protective species such as *F. prausnitzii* [34,51,52,53]. Vegans also appear to lack the intestinal microbiota for converting dietary l-carnitine into the proatherosclerotic TMAO [57]. Reduced levels of inflammation may be the key feature linking the vegan gut microbiota with protective health benefits. The role of dietary fiber in promoting lower levels of inflammation in subjects following a vegan diet warrants further exploration. The influence of plant *versus* animal protein sources on microbial profiles, metabolic syndrome and inflammation may be an avenue for future research as well.

New research holds great promise in revealing the mechanisms linking dietary patterns with gut microbiota profiles, obesity, inflammation, and disease states. For example, recent studies have revealed the link between inflammasomes, a group of protein complexes that recognize inflammation-inducing stimuli, and obesity, metabolic syndrome, insulin signaling and atherosclerosis [55,72,73,74]. Inflammasomes play a role in regulating gut microbiota and gut homeostasis, which in turn affects the immune homeostasis of the entire host organism [75]. The role of inflammasomes in regulating gut flora and the subsequent impact on metabolic syndrome is an exciting new field of study which may help elucidate the mechanism by which diet impacts gut microflora, inflammation and health.

An area in need of future research is the distinction between long-term diet adherence and short-term diet therapy. Wu *et al*. [22] postulate that long-term diets are responsible for the distinct enterotypes seen between those who follow an omnivorous diet and those who eat little or no animal products. Yet some studies have found that a dietary change can induce a partial shift in gut microbiota in a matter of weeks or even days [7,43]. It seems likely that sudden changes in diet, such as adopting a vegan diet to improve disease outcome, may alter the relative abundance of different taxa in the gut, without shifting the host into an entirely new enterotype [33]. As vegan diets are gaining in interest as medical nutrition therapy, it is important to discern the long-term advantage of short-term diet change. If patients need to maintain a strict vegan diet for long periods of time to reap continued health benefits, compliance may become an issue.

Diet compliance by patients is a critical factor in the feasibility of a vegan diet as adjuvant therapy in disease management. Barnard *et al*. [76] found that patients following a low-fat vegan diet for management of type 2 diabetes had diet compliance rates comparable to that of patients following a conventional diabetes diet after 74 weeks. Study participants adhering to the low-fat vegan diet demonstrated increased carbohydrate and fiber intake and decreased fat and cholesterol intake, suggesting that the diet has not only acceptability but applications for medical nutrition therapy. However, high drop-out rates are a concern in studies in which subjects are compelled to adopt a vegan or even a vegetarian diet [5]. While vegan diets have been shown to improve metabolic conditions in type 2 diabetes patients, similar improvements have been achieved with other diets including the Mediterranean diet, a low-carbohydrate/high-protein diet, and a vegetarian diet. Thus a patient’s personal taste and cultural traditions may need to dictate whether a vegan diet is the ideal choice for medical nutrition therapy [77].
